# Microscale Surface Tension and its Applications

**DOI:** 10.3390/mi10080526

**Published:** 2019-08-09

**Authors:** Pierre Lambert, Massimo Mastrangeli

**Affiliations:** 1 TIPs, Université Libre de Bruxelles, 1050 Bruxelles, Belgium; 2 ECTM, Delft University of Technology, 2628CT Delft, The Netherlands

**Keywords:** contact angle, droplets, liquid bridge, microfabrication, micromanipulation, pick-and-place, soft robotics, surface tension, wetting

More than 200 years since the earliest scientific investigations by Young, Laplace and Plateau, liquid surface tension is still the object of thriving fundamental and applied research. Partly inspired by nature’s evolutionary designs exploiting physical properties inherent to liquids, this research is enabling a rich and ever expanding set of technological applications. *Micromachines’* 2018 Special Issue on “Microscale Surface Tension and its Applications” was therefore conceived to present fundamental knowledge, showcase relevant ongoing works and highlight prospective research directions regarding capillarity, wetting, and collateral topics. 

Building on significant advances in miniaturization and soft matter, as well as in metrology and interfacial engineering, surface tension effects are indeed a major key to current developments in soft and fluidic microrobotics, precision micromanipulation and fluid/solid interactions. Benefiting from scaling laws, surface tension and capillary effects are expected to enable and support sensing, actuation, adhesion, confinement, compliance, and other structural and functional properties necessary in micro- and nanosystems. 

This Special Issue successfully gathered novel and multidisciplinary contributions on capillary micromechatronics (capillary grippers, vibration-induced transport of droplets, capillary actuators, self-alignment), superhydrophobic and self-lubricating surfaces, soluto-capillary Marangoni-based micromixing, and droplets micromanipulation. Worth highlighting are also two reviews on interfacial tension measurement and self-cleaning surfaces.

This varied and stimulating ensemble of contributions echoes many of the interests and directions identified during the *1st International Conference on Multiscale Applications of Surface Tension* (microMAST 2016), organized in September 2016 by the Belgian thematic network on Microfluidics and Micromanipulation (www.micromast2016.be). The goal of that conference and network was, and remains, to bring together various, interrelated or complementary research communities to collectively address up-to-date and unsolved questions concerning the broad field of surface tension effects.

In recapitulating the spirit of that ongoing enterprise, we hope that the interplay between fundamental questions and relevant applications driven by the downscaling of capillary effects captured in this Special Issue will provide an inspiring point of view for the readership of *Micromachines*.

